# Ordered Pt_3_Mn Intermetallic Nanoparticles Supported on Atomically Dispersed Mn–N–C as Electrocatalysts for Fuel Cells

**DOI:** 10.1002/anie.7818348

**Published:** 2026-06-09

**Authors:** Gongjin Chen, Tianshuai Wang, Xiaoyi Qiu, Shiyuan Liu, Cunpu Li, Zidong Wei, Wei Xing, Haijiang Wang, Minhua Shao

**Affiliations:** ^1^ Department of Chemical and Biological Engineering The Hong Kong University of Science and Technology Kowloon Hong Kong China; ^2^ Department of Mechanical and Energy Engineering Key Laboratory of Energy Conversion and Storage Technologies Southern University of Science and Technology Shenzhen Guangdong China; ^3^ CIAC‐HKUST Joint Laboratory for Hydrogen Energy The Hong Kong University of Science and Technology Kowloon Hong Kong China; ^4^ Energy Institute The Hong Kong University of Science and Technology Kowloon Hong Kong China; ^5^ Xi'an Key Laboratory of Functional Organic Porous Materials School of Chemistry and Chemical Engineering Northwestern Polytechnical University Xi'an China; ^6^ State Key Laboratory of Advanced Chemical Power Sources School of Chemistry and Chemical Engineering Chongqing University Chongqing China; ^7^ State Key Laboratory of Electroanalytical Chemistry Jilin Province Key Laboratory of Low Carbon Chemical Power Changchun Institute of Applied Chemistry Chinese Academy of Sciences Changchun China; ^8^ Guangzhou Key Laboratory of Electrochemical Energy Storage Technologies Fok Ying Tung Research Institute The Hong Kong University of Science and Technology Guangzhou China

**Keywords:** electrocatalysis, fuel cells, intermetallic compounds, oxygen reduction reaction, single‐atom

## Abstract

Compared with conventional solid‐solution alloy nanoparticles with disordered atomic structures, platinum (Pt)‐based intermetallic compounds (IMCs) are recognized as highly promising electrocatalysts for practical fuel cell applications, on account of their long‐range periodically ordered atomic arrangements. Nevertheless, the rational development of Pt‐based catalysts featuring both high intrinsic activity and long‐term durability remains a key challenge in this field. In this work, by simultaneously introducing manganese (Mn) with low‐electronegativity into both the active component and the support, we report an efficient electrocatalyst toward the oxygen reduction reaction (ORR), composed of L1_2_‐ordered Pt_3_Mn nanoparticles on Mn single‐atom nitrogen‐doped carbon support (L1_2_‐Pt_3_Mn@Mn–N–C). The incorporation of Mn, the strong anchoring effect arising from the hierarchically porous structure of the support, and the directional interfacial electron transfer between L1_2_‐Pt_3_Mn and Mn–N–C synergistically mitigate the adsorption strength of key oxygen intermediates and suppress the dissolution of surface Pt sites. Superior catalytic performance and durability are validated in proton exchange membrane fuel cells (PEMFCs), achieving a peak power density of 1.15 W cm^−2^ under H_2_/air conditions. After 30 000 square‐wave cycles, the voltage loss at 0.8 A cm^−2^ is only 19 mV, ranking it among the top‐performing Pt‐based cathode catalysts reported to date.

## Graphical abstract

Ordered L1_2_‐Pt_3_Mn intermetallic alloys are uniformly deposited onto atomically dispersed Mn–N–C supports. The synergistic effect between MnN_4_ sites and Pt_3_Mn elevates interfacial binding strength and dissolution barriers while favorably weakening *OH adsorption. It delivers exceptional catalytic activity and stability in fuel cells, serving as an ideal candidate for oxygen electroreduction.

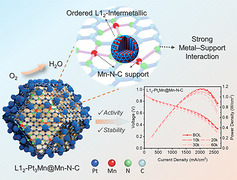

## 1 Introduction

Fuel cells, as a core technology for achieving carbon neutrality goals, are hindered by the high Pt demand and short lifespan of sluggish cathode ORR electrocatalysts [1, 2]. While Pt‐based alloys (PtM, M = transition metals) have significantly improved MA through ligand effects and lattice strain, issues like transition metal dissolution in disordered alloys and particle agglomeration during high‐temperature synthesis severely limit their durability [3, 4]. The cubical ordered IMC L1_2_‐Pt_3_M has stood out due to its long‐range atomic ordering and strong Pt‐M bonding characteristics in recent years [5]. However, these alloys inevitably suffer dealloying during voltage cycling in membrane electrode assemblies (MEAs), leading to significant losses in electrochemical active surface area (ECSA) and strain effects. Dissolved Co/Fe/Ni ions tend to undergo Fenton reactions, generating oxygen radicals and causing membrane degradation [6, 7]. Therefore, Mn, with the lowest electronegativity and lower Fenton reaction activity in the transition metals group, has received more attention [8, 9, 10]. The Fenton reaction rate constant of the Mn ion is approximately one order of magnitude lower than that of the Fe ion, which significantly reduces membrane degradation risk [11]. Apart from being more membrane‐friendly, PtMn alloys with lower electronegative metals inherently exhibit degradation‐resistant stability, effectively mitigating dealloying processes in acidic environments [12].

Both the alloy and the catalyst support need to exhibit excellent decomposition resistance and collaborative effects to withstand the harsh conditions of advanced MEAs. Many studies claim that the Pt‐based alloy particles often deposit on traditional carbon supports, such as Vulcan XC‐72 and Ketjen Black, making it challenging to secure ultrafine particles due to weak interactions with Pt‐based alloys, leading to nanoparticle migration, agglomeration, or detachment [13]. Nitrogen‐doped carbon‐supported single‐atom catalysts (M–N–C) have emerged as the ideal carriers for Pt‐based catalysts due to their unique electronic properties and structural advantages. The highly dispersed M–N_4_ sites in M–N–C not only provide abundant anchoring sites but also inhibit the relocation and accumulation of Pt nanoparticles through strong metal‐support interactions (SMSI) [14, 15]. Additionally, their hierarchical pore structure and high surface area promote mass transfer and reduce concentration polarization [16]. It is crucial to design high‐performance Pt‐based alloy catalysts from a holistic perspective, considering this dual regulation of the support and active components. Despite research on single‐atom carbon‐supported Pt‐based intermetallic catalysts, most studies rely on heterologous metal combinations, for example, Fe/Co/Mn single atoms with Pt–Co/Pt–Fe intermetallic, lacking unified electronic modulation from homologous metal species.

Herein, we introduce a highly efficient L1_2_‐Pt_3_Mn@Mn–N–C electrocatalyst for oxygen reduction, composed of an ordered L1_2_‐type Pt_3_Mn intermetallic alloy supported by Mn–N–C with abundant single‐atom sites. By precisely regulating the ordering and particle size distribution of Pt_3_Mn (∼3.5 nm) and leveraging the structural merits of the Mn–N–C support, this catalyst effectively inhibits the aggregation of Pt_3_Mn nanoparticles via the strong anchoring effect of Mn–N_4_ centers. This strategy significantly enhances the interfacial binding strength and dissolution resistance between Pt‐based species and the Mn–N–C, thereby simultaneously boosting activity and durability. Specifically, the robust Pt–Mn metallic bond and Pt‐C interfacial bond, coupled with the directional interfacial electron transfer from Mn–N_4_ to Pt clusters, synergistically weaken the adsorption strength of *OH (1.55 eV) and raise the Pt dissolution energy (1.53 eV). These favorable features are fully validated by systematic electrochemical characterizations and comprehensive MEA tests. In particular, electrochemical evaluations reveal that the MA decay rate is merely 16.1% after 30 000 potential cycles, far exceeding the stability of commercial Pt/C catalysts. In practical MEA tests with a total loading of 0.2 mg_Pt_ cm^−2^, the voltage decay at 0.8 A cm^−2^ is only 19 mV after 30 000 cycles, accompanied by an impressive MA retention of 63.7%, meeting the DOE 2025 targets and ranking among the most robust Pt‐based catalysts reported to date. Furthermore, the catalyst delivers outstanding stability over 100 h of operation under the harsh condition of 0.67 V in H_2_/air fuel cells. This work unveils the synergistic effect between Pt‐based nanoparticles and atomically dispersed MnN_4_‐enriched carbon supports, which may provide a rational strategy for designing high‐performance electrocatalysts for diverse electrochemical reactions.

## 2 Results and Discussion

### 2.1 Physicochemical Properties

**FIGURE 1:**
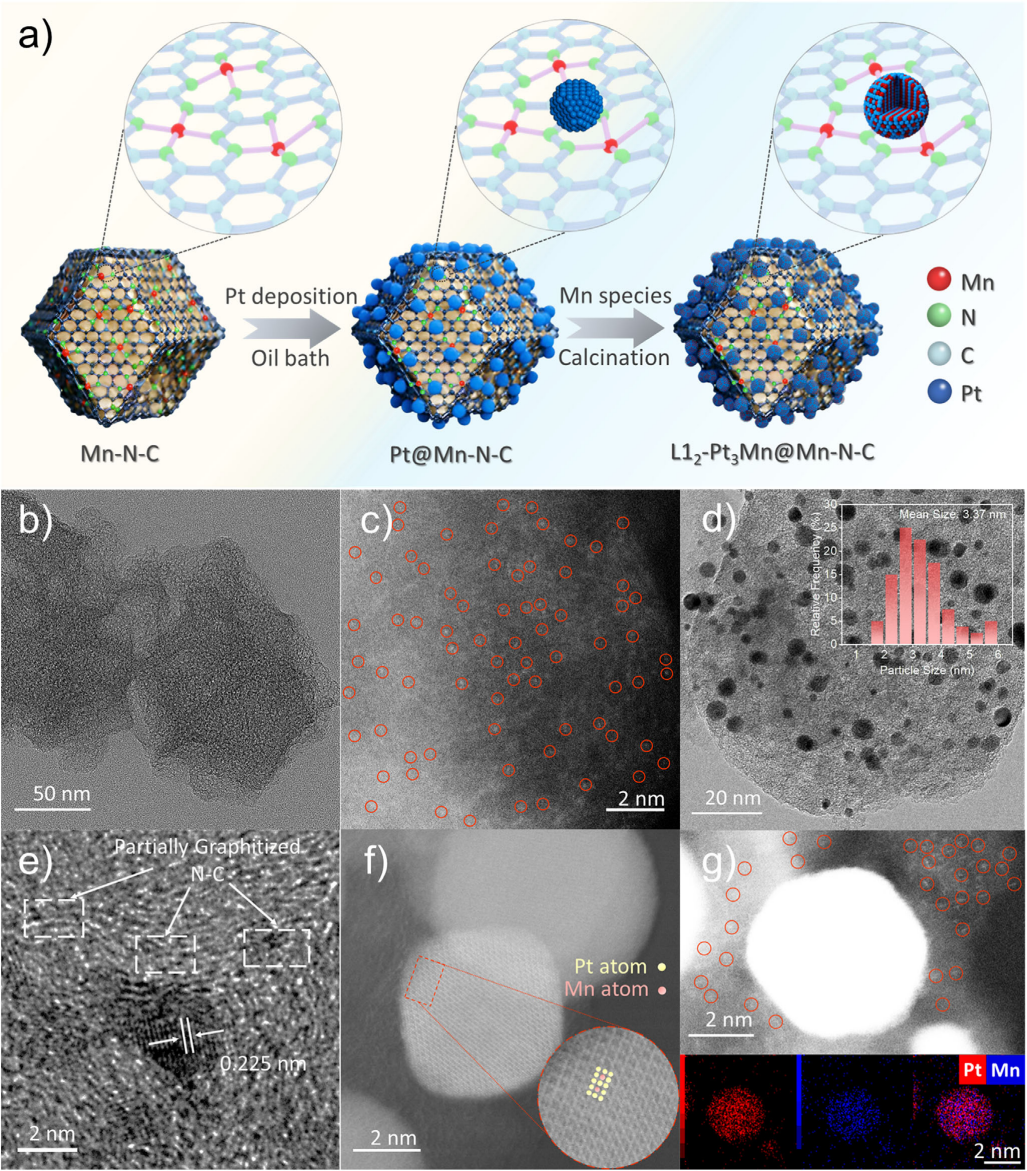
(a) Schematic illustration of the synthesis process for the L1_2_‐Pt_3_Mn@Mn–N–C catalyst. (b) TEM image, and (c) aberration‐corrected HAADF‐STEM of Mn–N–C. Structural characteristics of ordered L1_2_‐Pt_3_Mn@Mn–N–C: (d) TEM image, the inset image presents the associated particle size distribution, (e) high‐resolution TEM image, (f) atomically‐resolved HAADF‐STEM image with a magnified view with the rectangular area, and (g) representative HAADF‐STEM image (bright spots circled in red are ascribed to the Mn single atoms) along with the corresponding STEM‐EDS elemental mapping for Pt and Mn.

The L1_2_‐Pt_3_Mn@Mn–N–C catalyst is prepared via a stepwise impregnation‐reduction method (Figure 1a). First, Pt nanoparticles are deposited on the Mn–N–C support using ethylene glycol as the reducing agent to obtain the Pt@Mn–N–C, and then an additional Mn source is introduced to perform intermetallic alloying. The particle size of the Mn–N–C carbon support was precisely controlled to approximately 100 nm (Figure S1) to ensure suitable ionomer dispersion and mass transport within the catalyst layer [5, 17]. The carbonized Mn–N–C support retains an intact polyhedral structure with wrinkled edges (Figure 1b), and this unique morphology endows the material with abundant pore structures, which are beneficial for inhibiting the agglomeration of Pt nanoparticles during the deposition process. The HAADF‐STEM image in Figure 1c shows uniform bright dots, corresponding to automatically dispersed single atoms on the carbon substrate. The detailed synthetic procedure is illustrated in Figure S2, wherein a freeze‐drying strategy is introduced into the synthetic system to suppress the detachment of Pt nanoparticles, which provides an effective approach for subsequent size regulation of the ordered intermetallic compounds. The alloying process induces negligible change to the dodecahedral morphology of the Mn–N–C support (Figure S3). Similar to commercial Pt/C, both the pristine Pt and Pt_3_Mn nanoparticles are uniformly distributed on the Mn–N–C without obvious agglomeration (Figure S4), which is mainly attributed to the superiority of vacuum freeze‐drying over conventional oven drying, as this technique can firmly anchor the Mn source on the Pt@Mn–N–C during the solvent sublimation process. During vacuum freeze‐drying, the solvent directly sublimates, avoiding the nanoparticle agglomeration and uneven Mn source distribution caused by surface tension during liquid‐phase drying [18]. As shown in Figure 1d, Figures S5 and S6, the average particle sizes of commercial Pt/C, Pt@Mn–N–C and L1_2_‐Pt_3_Mn@Mn–N–C are 2.23, 2.59, and 3.37 nm, respectively. Among them, the ultrafine Pt nanoparticles are uniformly deposited on both the surface and inner pore channels of the Mn–N–C support. The wrinkled morphology and mesoporous structure of the support enable the physical confinement of Pt particles and can effectively alleviate their detachment and agglomeration during the ORR. Compared with Pt@Mn–N–C, L1_2_–Pt_3_Mn@Mn–N–C exhibits a slight increase in particle size, indicating that the nanoparticles undergo mild coarsening during the high‐temperature annealing process, while most of the obtained particle sizes fall within the optimal range (2–4 nm) for high‐performance ORR catalysts [19]. This can be ascribed to the synergistic effect between the Pt_3_Mn IMCs and Mn–N–C supports, which effectively mitigates the Ostwald ripening of the nanoparticles during the high‐temperature structural transformation process [20]. The high‐resolution TEM image in Figure 1e shows a lattice spacing of 2.25 Å, which matches well with the (111) crystal plane of L1_2_‐Pt_3_Mn. In addition, a partially graphitized carbon layer derived from the Mn–N–C support is observed around the IMC particles, which contributes to the electrical conductivity and stability of the catalyst [21]. Due to the difference in atomic mass, Pt and Mn atoms display distinct Z‐contrast in the HAADF‐STEM image. Figure 1f reveals that the brighter Pt atoms and the darker Mn atoms are arranged periodically, clearly indicating the formation of the L1_2_ intermetallic compound. Furthermore, the fast Fourier transform (FFT) pattern and the selected‐area electron diffraction (SAED) pattern further confirm the successful preparation of a highly ordered intermetallic structure (Figure S7). The HAADF‐STEM image demonstrates that abundant Mn‐N_4_ moieties are distributed around the L1_2_–Pt_3_Mn particles (Figure 1g), which is expected to strengthen the SMSI and thus optimize the catalytic performance. The corresponding EDS mapping proves that Pt and Mn are uniformly distributed inside the nanoparticles. Notably, the mapped area of Pt is faintly larger than that of Mn, suggesting the formation of a Pt‐rich shell on the alloy surface. This is a common feature of Pt‐based IMCs after high‐temperature treatment and can be attributed to the surface segregation of Pt atoms [22, 23]. The presence of this thin Pt‐rich shell not only exposes more active sites to enhance the activity but also suppresses the Mn dissolution during the ORR [24]. The ICP‐MS results are in good agreement with the STEM observations. The Pt and Mn contents in L1_2_‐Pt_3_Mn@Mn–N–C are 29.72 and 4.41 wt%, respectively (Table S1). After subtracting the amount of Mn in single‐atom sites (2.79 wt%), the atomic ratio of the remaining Mn to Pt is approximately 1:3, which is consistent with the theoretical ratio of L1_2_‐Pt_3_Mn. These results confirm that the L1_2_‐Pt_3_Mn with an atomic‐scale ordered structure is successfully prepared and uniformly distributed on the Mn–N–C support.

**FIGURE 2:**
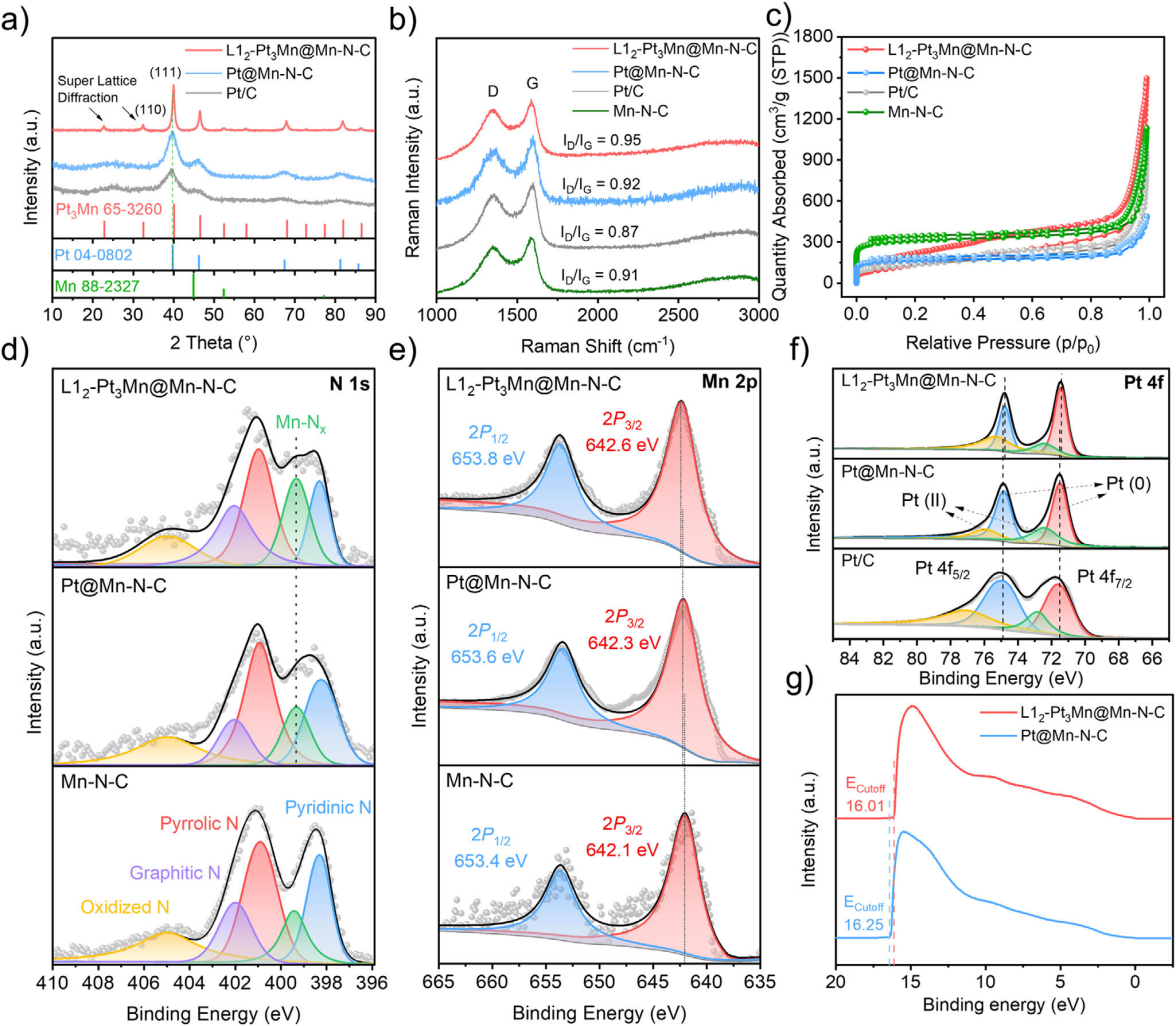
(a) XRD patterns, (b) Raman spectra, (c) the N_2_ adsorption–desorption isotherms, and XPS spectra of (d) N 1s, (e) Mn 2p, and (f) Pt 4f of L1_2_‐Pt_3_Mn@Mn–N–C, Pt@Mn–N–C, commercial Pt/C, and Mn–N–C catalysts. (g) UPS spectra of L1_2_‐Pt_3_Mn@Mn–N–C and Pt@Mn–N–C.

The crystal structure of as‐prepared catalysts is studied by XRD measurement. As shown in Figure 2a, the XRD pattern of Pt@Mn–N–C is highly analogous to commercial Pt/C, exhibiting only Pt‐related diffraction peaks without detectable impurity phases. These results confirm that the effect of the Mn–N–C support on the intrinsic Pt crystal structure is negligible. In contrast, the XRD pattern of L1_2_‐Pt_3_Mn@Mn–N–C clearly displays Pt_3_Mn diffraction peaks, particularly the (110) peak at 32°, which can be assigned to the typical structure of ordered L1_2_‐Pt_3_Mn nanoparticle (PDF #65‐3260). This observation verifies the successful formation of the L1_2_ intermetallic phase, which is in good agreement with the HAADF‐STEM results. The diffraction peaks of the Pt_3_Mn catalyst exhibit a slight shift toward higher 2‐theta angles, attributed to lattice contraction from regular atomic ordering. Furthermore, the significant electronegativity difference between Pt and Mn enables stronger metal bonds, resulting in shortened bond lengths [25]. This feature is presumably beneficial for enhancing catalytic stability. Figure S8 presents the XRD patterns of L1_2_–Pt_3_Mn@Mn–N–C annealed at 500°C to 900°C, revealing regular changes in the types and relative intensities of diffraction peaks with increasing temperature. At pyrolysis temperatures ≥ 700°C, all samples exhibit superlattice peaks, for example, (100), (110), and (210), alongside intermetallic peaks. The order degree of alloy nanoparticles is evaluated by the integrated area ratio of the (110) and (111) peaks [26, 27]. Quantitative calculations show that the proportion of the 750°C pyrolyzed sample is closest to the theoretical value (Figure S9). Above 750°C, the order degree tends to stabilize, while XRD peaks become increasingly sharp and narrow, suggesting gradual alloy nanoparticle growth. Similarly, systematic XRD comparisons were conducted to assess the influence of pyrolysis time (Figure S10). Results demonstrate that ≥ 2 h are required to form a stable intermetallic phase. Notably, excessively high temperatures or prolonged times exacerbate alloy agglomeration. Thus, pyrolysis at 750°C for 2 h is identified as the optimal synthesis condition, balancing particle size and the crystallinity of the L1_2_ intermetallic phase. Figure 2b displays the Raman spectra of all catalysts, with characteristic bands at ∼1340 cm^−1^ (D band) and 1590 cm^−1^ (G band). Specifically, L1_2_‐Pt_3_Mn@Mn–N–C catalyst exhibits a higher I_D_/I_G_ ratio than commercial Pt/C, revealing higher defect density in the Mn–N–C support. This structural feature not only offers abundant anchoring sites for Pt_3_Mn nanoparticles and effectively inhibits their agglomeration but also induces the formation of a rich micro/mesoporous structure to enhance mass transfer efficiency. Meanwhile, the abundant defects and Mn–N_4_ moieties in the carbon support are favorable for constructing a SMSI with the Pt_3_Mn nanoparticles, which further stabilizes the catalytically active sites [28]. Moreover, the I_D_/I_G_ undergoes a minor change during the transition from pure Mn–N–C support to Pt loading and Pt_3_Mn alloying. This confirms the structural integrity of the Mn–N–C framework remains intact during synthesis. The pore structure was characterized by BET measurements. As shown in Figure 2c, all samples display type IV adsorption isotherms, a typical feature of mesoporous materials. The pristine Mn–N–C sample retains a well‐developed porous structure with a high specific surface area (SSA) of 1256 m^2^ g^−1^, and its abundant mesopores help anchor Pt/Pt_3_Mn nanoparticles and accelerate mass transfer [29]. Compared to Pt/C, Mn–N–C supported catalysts possess larger SSA and mesopore volume (Figure S11 and Table S2). After depositing Pt‐based nanoparticles, the SSA of Pt@Mn–N–C and L1_2_–Pt_3_Mn@Mn–N–C catalysts decreased to 624 and 648 m^2^ g^−1^, respectively, with a significant reduction in macropore volume. These phenomena prove that Pt‐based nanoparticles are mainly loaded in the macropores of Mn–N–C support. In contrast, mesopore volume does not decrease but slightly increases, which is believed to arise from carbon framework reconstruction induced by the interaction between the Pt/Pt_3_Mn nanoparticles and the MnN_4_ sites of the Mn–N–C support during pyrolysis [30].

XPS is employed to characterize the surface chemical properties of the catalysts. The wide survey spectrum (Figure S12) clearly shows the presence of Pt, Mn, and N elements, verifying their successful incorporation. Table S3 presents that the Pt/Mn atomic ratio in L1_2_–Pt_3_Mn@Mn–N–C is close to 3:1, further proving the successful synthesis of Pt_3_Mn alloy. All samples exhibit nearly identical C 1s spectra and carbon contents (Figure S13), indicating that the synthetic strategy preserves the dominant surface chemical states of the carbon support. As shown in Figure 2d, the high‐resolution N 1s spectra demonstrate that the three catalysts share an identical nitrogen speciation, including oxidized N, graphitic N, pyridinic N, and Mn–N_x_. This indirectly validates that Pt_3_Mn formation does not damage the MnN_4_ sites in the Mn–N–C support. After Pt deposition, the pyridinic N peak intensity in Pt@Mn–N–C decreases significantly, accompanied by a small increase in Mn–N_x_. This is attributed to the preferential coordination between Pt atoms and pyridinic N species, while electron transfer between Pt and Mn enhances the structural stability of Mn–N_x_ moieties [31]. After alloying Pt_3_Mn, the integrated area of Mn–N_x_ further enhances and becomes the dominant coordination structure. This signifies the strong interactions between Pt/Mn atoms and N species, which is expected to boost ORR activity and stability [15]. In the Mn–N–C support, Mn atoms stably exist as Mn–N_x_ peak by bonding with N atoms in the carbon substrate, and the Mn 2P_3/2_ peaks appears in the relatively low binding energy region (Figure 2e). Upon introducing Pt to form Pt@Mn–N–C, the Mn 2P_3/2_ peak positively shifts to 642.3 eV relative to Mn–N–C, originating from electronic interactions between Pt and Mn atoms. This electron transfer assists in stabilizing Mn in a higher valence state, thereby preliminarily enhancing the Mn–support interfacial binding interaction [32]. The more pronounced positive shift in L1_2_‐Pt_3_Mn@Mn–N–C indicates that the Pt_3_Mn formation induces stronger covalent interactions. This not only drastically elevates the Mn binding energy relative to that in Mn–N–C but also stabilizes high‐valence Mn species by enhancing the covalency of Mn–N bonds, thereby effectively suppressing Mn dissolution during electrochemical cycling and providing structural assurance for the catalyst's long‐term stability. Figure 2f illustrates the Pt 4f spectra, revealing that surface Pt species in the samples are predominantly metallic. Compared with commercial Pt/C, both Mn–N–C supported catalysts exhibit a negative shift in Pt binding energy, demonstrating that MnN_4_ sites favor strengthening the metal‐support interactions [33]. Specifically, the Pt 4f_7/2_ peak of Pt@Mn–N–C shows a trivial negative shift, arising from the Mn–N–C support's coordination effect and electron transfer that regulates Pt's electronic environment. Since the electronegativity of Mn (1.55) is lower than that of Pt (2.28), electrons transfer from Mn to Pt, increasing the electron density of Pt. Meanwhile, the N atoms in the support modulate the electronic structure of the surrounding C/N matrix to enhance the interactions with Pt. These two factors collectively reduce the Pt binding energy. The more negative shift of the Pt 4f peak in L1_2_–Pt_3_Mn@Mn–N–C proves that Pt_3_Mn alloying triggers stronger electronic interactions. Alloying amplifies the electron‐donating effect from Mn to Pt, together with the synergistic regulation of MnN_4_ sites in the support, remarkably elevating the Pt electron density [34]. These dual effects of alloying and N‐site synergy are responsible for the more pronounced electronic structure variation. A lower Pt 4f binding energy corresponds to a downshift of the Pt d‐band center, weakening the adsorption of oxygen intermediates (e.g., O*, OH*) on the Pt surface, thereby boosting the catalytic efficiency of active sites [35]. Furthermore, the effect of annealing temperature on the Pt 4f and Mn 2p binding energies is analyzed (Figure S14). As the temperature increases, Pt binding energy first decreases and then stabilizes at 750°C, reflecting an increase in Pt electron density with a higher alloying degree. This variation directly correlates with electrocatalytic activity. In contrast, Mn 2p binding energy displays an opposite trend, indicating the transformation of the Mn electronic environment from N‐coordination‐dominated to Pt‐Mn alloying‐dominated. As the convergence point, pyrolysis at 750°C endows Pt and Mn with an optimal electronic structure, balancing coordination stability and alloying effect. To clarify the catalyst's work function and evaluate electron transfer characteristics, UPS measurements are performed (Figure 2g and Figure S15). UPS results show that the secondary electron cutoff energy (*E*_cutoff_) decreases from 16.35 eV (Pt@Mn–N–C) to 16.1 eV (L1_2_‐Pt_3_Mn@Mn–N–C), possessing a higher work function (*Φ* = 21.22 eV‐E_cutoff_). It has been reported that for Pt‐based alloy catalysts, a higher work function corresponds to a lower d‐band center [36], which is consistent with the XPS analysis. The downshift of L1_2_‐Pt_3_Mn@Mn–N–C's *d*‐band center not only facilitates the rapid desorption of reaction intermediates to release active sites but also moderately tunes the O_2_ activation ability, thus establishing a more favorable ORR kinetic environment [37].

### 2.2 Electrochemical Properties

**FIGURE 3:**
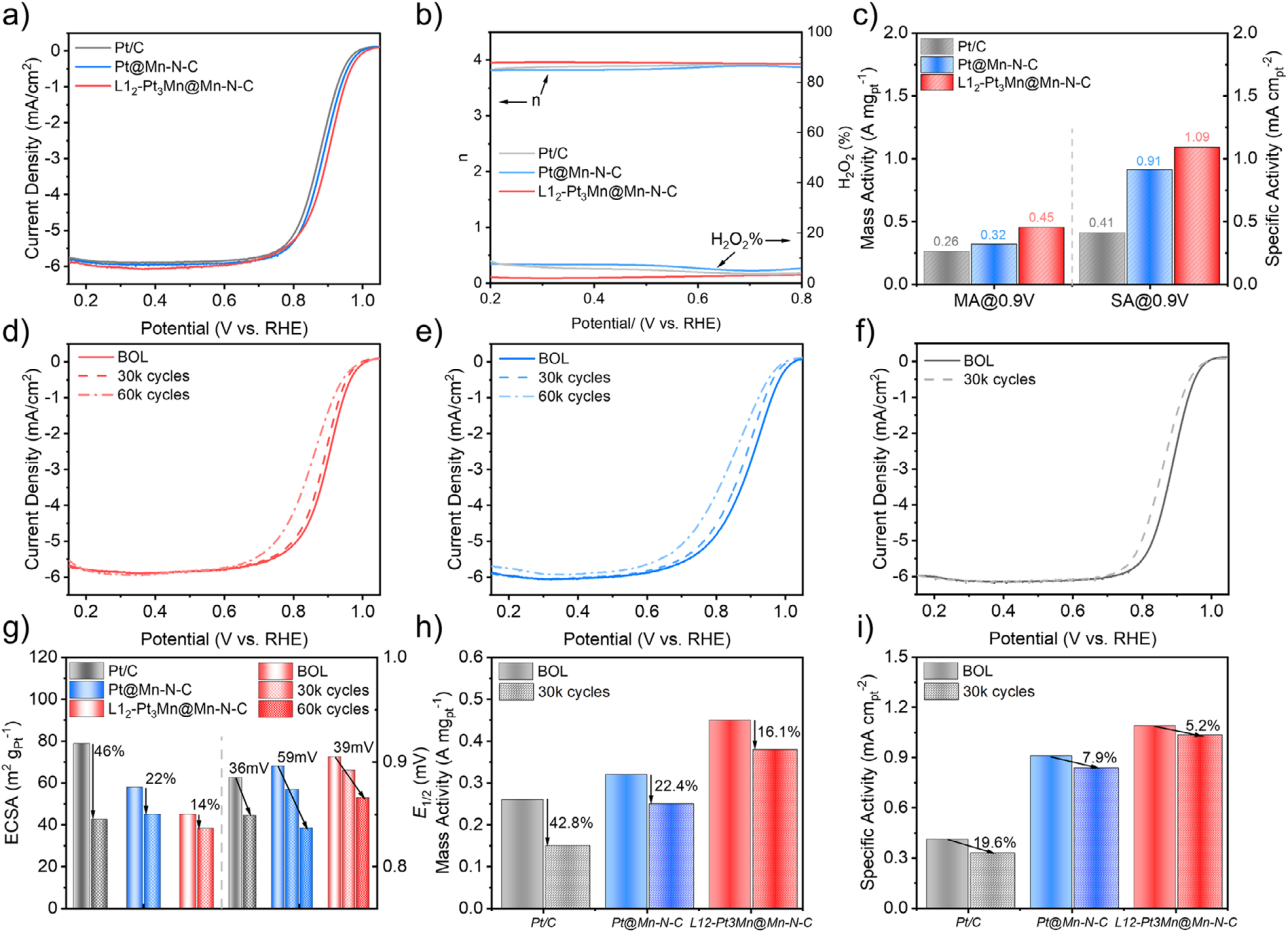
Electrochemical measurements of the L1_2_–Pt_3_Mn@Mn–N–C, Pt@Mn–N–C, and commercial Pt/C. (a) Steady state LSV curves and (b) electron transfer number (*n*) and H_2_O_2_ yield in 0.1 M HClO_4_ solution, while (c) presents the corresponding MA and specific activity (SA) at 0.9 V. Steady‐state ORR polarization before and after 30 or 60 k accelerated stress tests (AST) via square‐wave potential cycling from 0.6 to 0.95 V in O_2_‐saturated 0.1 M HClO_4_ solution: (d) L1_2_–Pt_3_Mn@Mn–N–C, (e) Pt@Mn–N–C, and (f) commercial Pt/C. Variations of (g) ECSA and E_1/2_, (h) MA, and (i) SA before and after the AST test.

To evaluate the electrochemical performance, systematic rotating disk electrode (RDE) tests are performed on L1_2_–Pt_3_Mn@Mn–N–C, Pt@Mn–N–C, and commercial Pt/C in 0.1 M HClO_4_ at room temperature. As shown in Figure 3a, with comparable limiting current densities, the combination of Pt_3_Mn and Mn–N–C delivers the highest E_1/2_ (0.905 V), showcasing excellent oxygen reduction performance. Figure S16 shows the Tafel plots of these catalysts. The lower Tafel slopes of both Mn–N–C catalysts compared to commercial Pt/C suggest that moderate Mn‐induced strain modulation effectively enhances charge transfer kinetics. Rotating ring‐disk electrode (RRDE) measurements reveal that the H_2_O_2_ yield of L1_2_–Pt_3_Mn@Mn–N–C remains below 3% (Figure 3b and Figure S17), confirming a highly selective four‐electron ORR pathway. Furthermore, the lower H_2_O_2_ yield relative to Pt@Mn–N–C reflects a more favorable 4 e^−^ pathway and superior catalytic selectivity. For intrinsic activity evaluation, Figure 3c presents the MA and SA at 0.9 V, respectively. L1_2_–Pt_3_Mn@Mn–N–C outperforms both Pt@Mn–N–C and Pt/C at these potentials, demonstrating that its exceptional performance arises from the constructive interaction between the active component and the support. This behavior can be ascribed to the distinct atomic size difference between Mn (132 pm) and Pt (139 pm), which introduces compressive strain on surface Pt atoms in the Pt_3_Mn intermetallic compound [38]. Such compressive strain weakens oxygen adsorption energy, thereby improving ORR activity. Accordingly, the activity follows the sequence, L1_2_–Pt_3_Mn@Mn–N–C > Pt@Mn–N–C > commercial Pt/C, which is consistent with the negative shift trend observed in Pt 4f XPS spectra. Figure S18 summarizes the cyclic voltammetry (CV) and LSV curves of L1_2_–Pt_3_Mn@Mn–N–C annealed at different temperatures. The catalyst annealed at 750°C exhibits the highest E_1/2_, matching the optimal balance between atomic ordering and particle size revealed by XRD. This further establishes the clear structure‐activity relationship between ordering degree and electrocatalytic activity.

Systematic square‐wave potential cycling‐based AST measurements are conducted on the three catalysts, with corresponding LSV and CV curves displayed in Figure 3d–f and Figure S19. For the L1_2_–Pt_3_Mn catalyst, the H adsorption/desorption and oxygen reduction peaks exhibit a positive shift relative to Pt/C, proving a downshifted *d*‑band center and weaker binding of oxygenated intermediates on Pt active sites, which is consistent with the XPS observations. The ECSA of the as‐obtained catalysts and Pt/C is estimated from hydrogen underpotential deposition/stripping (*H*_upd_) curves (Figure 3g). Commercial Pt/C exhibits a higher initial ECSA than the other two catalysts. In contrast, the larger particle sizes of the as‐prepared catalysts, resulting from alloying and pyrolysis, lead to a reduced active area. However, after 30 000 cycles, the ECSA values of the three catalysts tend to converge to a comparable level. This demonstrates the superior stability of L1_2_–Pt_3_Mn@Mn–N–C, which is 60% of the loss observed for Pt@Mn–N–C and 30% of the loss for Pt/C. Results in Figure S19 reveal that the CV curves of two Mn–N–C supported catalysts show no obvious changes after 30 000 cycles, in contrast to commercial Pt/C. Furthermore, Figure 3g compares E_1/2_ degradation, and the potential attenuation of L1_2_‐Pt_3_Mn@Mn–N–C after 60 000 cycles (39 mV) is close to commercial Pt/C after 30 000 cycles (36 mV), which confirms the Mn–N–C support's critical role in enhancing stability. Figure 3h,i present the variations in MA and SA at 0.9 V, with L1_2_‐Pt_3_Mn@Mn–N–C retaining ultrahigh MA and SA retention rates of 83.9% and 94.8%, respectively, which are 1.8 and 1.2 times those of Pt/C. Figure S20 shows the XPS spectra after 30 000 cycles; the Pt 4f of L1_2_–Pt_3_Mn@Mn–N–C exhibits a positive shift relative to the initial state, with a marginal binding energy increase. This can be attributed to altered surface Pt electronic structure or surface Pt oxidation. In contrast, Mn 2p and N 1s spectra show negligible changes before and after the test, with the Mn–N_x_ peak well preserved. Additionally, XPS elemental analysis (Table S4) proves that the Pt:Mn atomic ratio remains approximately 3:1 after the AST, further verifying L1_2_–Pt_3_Mn@Mn–N–C's structural stability. Therefore, L1_2_–Pt_3_Mn@Mn–N–C and Pt@Mn–N–C are more durable than commercial Pt/C, with alloying further enhancing the electrochemical stability. The in situ metal dissolution behavior of the electrocatalysts was monitored using a coupled fixed‐probe RDE and ICP‐MS, at constant Pt loading on the electrodes. The calibration curves exhibit a good linear relationship between Pt/Mn concentrations and signal intensities (Figure S21). Figure S22 presents the Pt dissolution rates (ppb s^−1^) of the four catalysts during potential scanning. The Pt dissolution curves share an analogous trend but differ significantly in their maximum transient dissolution rates. In the 0.05 to 1.2 V potential window, L1_2_–Pt_3_Mn@Mn–N–C clarifies a peak dissolution rate of 1.7 ppb s^−1^, which is marginally lower than that of Pt@Mn–N–C (2.4 ppb s^−1^), much lower than that of Pt/C (3.2 ppb s^−1^), and also distinctly lower than the PtCo/C alloy catalyst (2.25 ppb s^−1^). These results demonstrate that the Mn–N–C support can effectively suppress Pt dissolution during redox processes, with the Pt_3_Mn intermetallic alloy further mitigating Pt loss. Figure S23 displays the transient Mn leaching amount under the same conditions, which is negligible compared to Pt. As compared to Table S5, L1_2_–Pt_3_Mn@Mn–N–C exhibits highly competitive ORR activity and durability among the reported binary and ternary Pt‐based catalysts.

### 2.3 Reaction and Formation Mechanism Analysis

**FIGURE 4:**
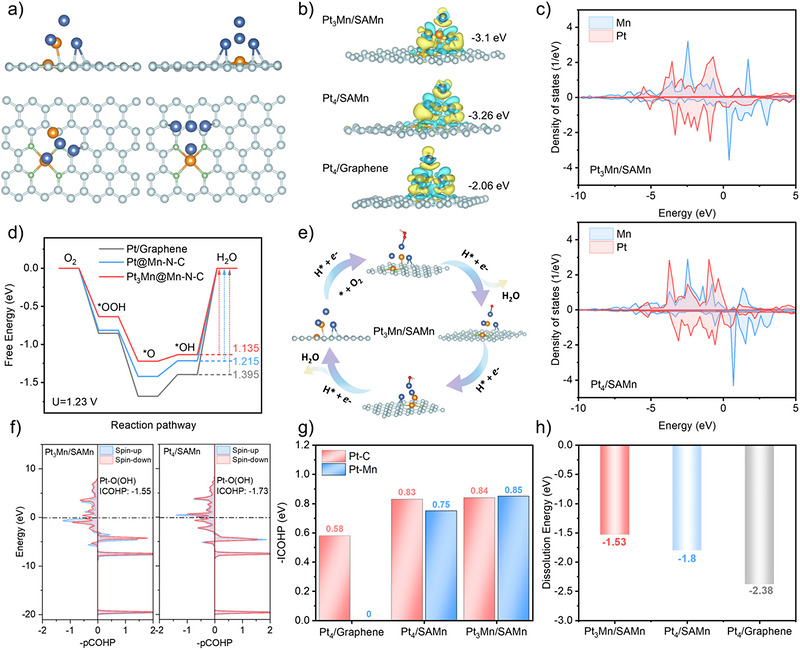
DFT calculations regarding the oxygen reduction reaction: (a) Atomic configurations of proximal Pt_3_Mn/SAMn and Pt_4_/SAMn. (b) Charge density difference analysis and corresponding binding energy. (c) Projected density of states (PDOS) of Pt and Mn. (d) Free energy diagrams depicting Pt_3_Mn/SAMn, Pt_4_/SAMn, and Pt_4_/Graphene at *U* = 1.23 V (vs. RHE), respectively. (e) Schematic of the ORR reaction pathway on Pt_3_Mn/SAMn. The gray, gold, blue, green, red, and white represent the C, Mn, Pt, N, H, and O atoms, respectively. (f) Crystal orbital Hamilton population (COHP) analysis of Pt‐O(OH) bonds. (g) Integrated COHP (ICOHP) of Pt–C and Pt–Mn bonds. (h) Dissolution energy for surface Pt atom for Pt_3_Mn/SAMn, Pt_4_/SAMn, and Pt_4_/Graphene models.

To unravel the underlying mechanisms accounting for the exceptional ORR catalytic activity and durability of the as‐prepared catalysts, DFT calculations were performed in this study. Theoretical models were constructed by depositing Pt_4_ and Mn‐substituted Pt_3_Mn clusters onto a Mn–N_4_ single‐atom carbon support (SAMn) and a pristine graphene support, respectively. Figure S24 presents the distinct loading configurations of the metal particles, considering both proximal and distal distributions of the nanoparticles relative to the single‐atom sites during the deposition process. Based on the calculated total energies (Table S6), both Pt_3_Mn and Pt_4_ clusters preferentially bind at the Mn‐N_4_ center rather than the pristine carbon rings (Figure 4a). The lower total energy indicates a thermodynamically more stable structure, confirming that the Mn single‐atom sites serve as specific and strong anchoring sites for Pt‐based clusters. Binding energy calculations further quantify the interfacial anchoring strength (Figure S25). The binding energies of Pt_3_Mn/SAMn and Pt_4_/SAMn reach −3.10 and −3.26 eV, which are significantly more negative than the value of −2.06 eV for the reference sample. These results thermodynamically validate the strong immobilization effect of the Mn–N–C support toward Pt‐based nanoparticles, which is distinctly superior to that of pure carbon support. The charge density difference presented in Figure 4b visually illustrates the interfacial charge transfer and redistribution between the Pt‐based clusters and the supports. For the Pt_4_/Graphene system, only weak and discrete charge transfer occurs at the contact interface between the clusters and the carbon support, without obvious directional electron migration. In sharp contrast, when Mn–N–C is employed as the support, significant and continuous charge rearrangement takes place at the interface between the Mn‐N_4_ sites and both Pt_3_Mn and Pt_4_ clusters. Electrons are evidently transferred from the support to the Pt‐based particles, accompanied by strong local electronic hybridization and redistribution between Pt and Mn atoms, thus verifying the formation of a pronounced SMSI. PDOS analysis further elucidates the microscopic origin of the strong anchoring effect and electronic modulation (Figure 4c). Near the Fermi level (0 eV), the electronic states of Pt and Mn exhibit significant orbital overlaps, confirming a strong electronic interaction between the two species. This interaction not only strengthens the binding between the clusters and the substrate but also tailors the electronic structure of the Pt active sites.

Figure 4d displays the ORR free energy diagrams of the three catalysts, where the rate‐determining step (RDS) is identified as the desorption of the *OH intermediate. Notably, the RDS energy of Pt_3_Mn/SAMn is merely 1.135 eV (Table S7), which is considerably lower than those of the other two catalysts. This indicates that the introduction of Mn single‐atom sites can effectively reduce the reaction energy barrier, thereby enhancing the catalytic efficiency of ORR. Figure 4e and Figure S26 illustrate the four‐electron ORR reaction pathways on the surfaces of the three catalysts. After protonation, O_2_ first forms *OOH on top of the surface Pt atoms. The *OOH then dissociates into *O and *OH, and the *OH undergoes further protonation to produce liquid water. COHP bonding analysis explains the intrinsic mechanism underlying the enhanced activity of L1_2_–Pt_3_Mn@Mn–N–C at the microscopic level (Figure 4f). For Pt_3_Mn/SAMn, the ICOHP value for the bonding between Pt and key ORR intermediates *O(OH) is −1.55 eV, which is more favorable than the corresponding values of −1.73 eV for Pt_4_/SAMn and −1.76 eV for Pt_4_/Graphene (Figure S27). This indicates that Pt_3_Mn/SAMn exhibits moderate adsorption strength toward intermediates, achieving an optimal adsorption‐desorption balance, which serves as the core reason for its improved catalytic activity. Furthermore, as the central active sites for oxygen reduction, the structural stability of Pt species can be verified by bonding strength and metal dissolution resistance. Figure 4g presents that the ICOHP values of Pt‐C bonds for the two Mn–N–C‐supported catalysts are 0.83 and 0.84 eV, respectively. Both are substantially higher than 0.58 eV for Pt_4_/Graphene, suggesting that the introduction of Mn‐N_4_ sites significantly enhances the interfacial bonding between Pt‐based clusters and the carbon substrate. Meanwhile, a stronger Pt‐Mn metallic bond is formed in L1_2_–Pt_3_Mn@Mn–N–C compared to Pt@Mn–N–C, indicating that the leaching of Mn atoms in the system is effectively suppressed. This inhibition effect facilitates the maintenance of structural integrity under practical electrocatalytic operating conditions. Typically, Pt dissolution is one of the major routes leading to the degradation of ORR catalysts [3]. The calculated surface Pt dissolution energies quantitatively evaluate the dissolution resistance of the catalysts (Figure 4h). Among the studied systems, Pt_3_Mn/SAMn exhibits the weakest tendency for surface Pt leaching, which corresponds to its optimal electrochemical dissolution resistance. This result is consistent with the in situ ICP‐MS measurements, providing robust thermodynamic assurance for the long‐term service of the catalyst under fuel cell operation.

### 2.4 Fuel Cell Tests

**FIGURE 5:**
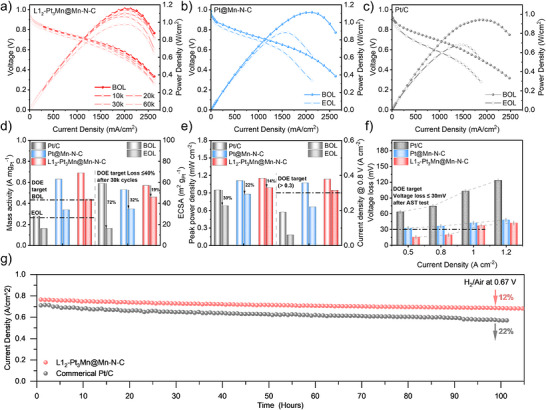
Fuel cell measurements of the as‐prepared electrocatalyst. H_2_‐Air fuel cell AST test results of the MEAs prepared by the cathode catalyst of (a) L1_2_–Pt_3_Mn@Mn–N–C, (b) Pt@Mn–N–C, and (c) commercial Pt/C, respectively, after various numbers of 0.6 to 0.95 V square wave cycles. All the fuel cell tests were performed with anode and cathode loadings of 0.05 and 0.15 mg_Pt_ cm^−2^ respectively, at 80°C with a 100% relative humidity and a 150 kPa back pressure in a 5 cm^2^ single PEM fuel cell. Variations of (d) MA at 0.9 V in H_2_‐O_2_ condition and ECSA, (e) peak power densities and current densities at 0.8 V in H_2_‐Air condition for L1_2_‐Pt_3_Mn@Mn–N–C, Pt@Mn–N–C, and commercial Pt/C before and after the AST tests. (f) Voltage losses at different current densities after AST test in H_2_‐Air condition. (g) Long‐term fuel cell steady‐state tests for L1_2_‐Pt_3_Mn@Mn–N–C and commercial Pt/C catalysts under H_2_‐Air conditions at a constant potential of 0.67 V.

A series of fuel cell tests are conducted on L1_2_‐Pt_3_Mn@Mn–N–C to verify its performance under real operating conditions. Optimizing the ionomer‐to‐carbon (I/C) ratio enabled L1_2_–Pt_3_Mn@Mn–N–C to attain high power density and energy efficiency in MEA. The appropriate ratio helps to balance proton conduction, active site exposure, and mass transport, minimizing voltage loss in the medium‐to‐high current density region [39]. Combined with polarization curves and EIS results (Figure S28), the optimal performance is obtained at an I/C ratio of 0.8. Besides the I/C ratio, humidity sensitivity is equally critical for catalysts, given that heavy‐duty scenarios often demand low‐humidity operation [40]. Figure S29 illustrates the MEA performance of L1_2_–Pt_3_Mn@Mn–N–C at different relative humidity (RH). No significant performance degradation in the high current density region occurs until the humidity decreases to 60% RH, underscoring the Mn–N–C support's excellent mass transfer capability. Square‐wave durability tests are operated on the as‐prepared catalysts and commercial Pt/C following the DOE protocol. Figure 5a–c display that L1_2_–Pt_3_Mn@Mn–N–C achieves a peak power density of 1.15 W cm^−2^, retaining 82% of its performance even after 60 000 cycles. Consistent with the RDE results, this proves the good scalability from half‐cell to full‐cell tests. Compared with commercial Pt/C, both Mn–N–C supported catalysts show smaller attenuation in ohmic and mass transport losses after 30 000 cycles. This is attributed to the inherent stability of Mn–N–C support, robust anchoring of active species, and enhanced cathode water management. EIS is employed to analyze the MEA's charge transfer resistance (*R*_ct_) at different current densities before and after AST. Impedance data at 0.1 A cm^−2^ can directly reflect the surface catalytic activity of the catalyst. Results show that L1_2_‐Pt_3_Mn@Mn–N–C has a smaller semicircle diameter (Figure S30 and Table S8), proving faster electron transfer kinetics and thus more favorable intrinsic ORR dynamics. After 30 000 cycles, the intermetallic alloy catalyst maintains a smaller *R*_ct_ value, demonstrating well‐preserved kinetic activity. At a high current density of 1.5 A cm^−2^ (Figure S31 and Table S9), two Mn–N–C supported catalysts exhibit slightly smaller low‐frequency arcs, indicating less resistance to mass transport in their electrodes than Pt/C. Previous reports mentioned that boosted transition metal ion concentration in the MEA ionomer phase may elevate local oxygen transport resistance [41]. In situ ICP‐MS and DFT calculations confirm that the anchoring effect in L1_2_–Pt_3_Mn@Mn–N–C mitigates Mn leaching, thereby reducing oxygen transport resistance and promoting high current density performance. Figure 5d summarizes the key performance indicators of catalysts after 30 000 AST cycles. The MA of L1_2_‐Pt_3_Mn@Mn–N–C meets the DOE target both before and after testing, approximately three times that of commercial Pt/C. Notably, the catalyst exhibited a marked difference in MA retention after 30 000 cycles between RDE and MEA tests. This arises from the MEA's more complex mass transfer, inevitable three‐phase interface degradation, and ionomer depolymerization, leading to more severe performance attenuation in the MEA [42]. The as‐synthesized catalysts exhibit lower initial ECSA than Pt/C, likely due to the larger particle size. However, the end‐of‐life ECSA of both Mn–N–C supported catalysts meet the DOE target, benefiting from the mesoporous Mn–N–C support, which efficiently alleviates particle agglomeration and loss during cycling. In practical H_2_‐Air fuel cells, the L1_2_‐Pt_3_Mn@Mn–N–C based MEA shows only 14% peak power density decay after 30 000 AST cycles (Figure 5e), representing excellent durability. In contrast, the power densities of Pt@Mn–N–C and Pt/C decrease by 22% and 30%, respectively. Particularly, L1_2_‐Pt_3_Mn@Mn–N–C retains a current density of 0.32 A at 0.8 V after 30 000 cycles, satisfying the DOE criterion and revealing that the strong interaction between Pt and Mn can withstand the harsh device‐level operating conditions. The DOE target specifies a voltage loss of ≤ 30 mV at 0.8 A cm^−2^, and L1_2_‐Pt_3_Mn@Mn–N–C exhibits a voltage loss of only 21 mV under H_2_‐Air conditions (Figure 5f).

STEM coupled with elemental analysis is employed to characterize the cathode catalyst and catalyst layer of the MEA after 30 000 cycles. As shown in Figure S32, the L1_2_‐Pt_3_Mn nanoparticles remain uniformly distributed on the Mn–N–C support, with a slight migration toward the support surface. The unharmed overall morphology of the Mn–N–C support after the aging test demonstrates its excellent mechanical stability. Statistical analysis reveals that the average nanoparticle size of L1_2_‐Pt_3_Mn@Mn–N–C slightly increases from 3.37 nm to 4.6 nm (Figure S33a), without the severe agglomeration commonly observed in analogous systems and commercial Pt/C (growth rates of ∼150%–200%) [43]. This further underscores the superiority of the Mn–N–C support in preserving nanoparticle size stability and suppressing Ostwald ripening. The post‐cycling XRD patterns (Figure S33b) exhibit diffraction peaks highly consistent with those of the pristine state, along with noticeably sharpened peak profiles, verifying the retention of the IMC crystal structure and corroborating the observed increase in particle size. The high‐resolution TEM image in Figure S33c shows clear lattice fringes assigned to the (111) plane. The SAED pattern also displays continuous, well‐resolved diffraction rings consistent with the characteristic features of ordered L1_2_‐Pt_3_Mn (Figure S33d). Furthermore, the HAADF‐STEM image confirms the well‐preserved Mn single‐atom sites after AST, and the corresponding elemental mapping demonstrates the homogeneous distribution of Pt and Mn throughout the catalyst, providing direct evidence for the structural integrity of the intermetallic phase as well as the structural robustness of the Mn–N–C support (Figure S34). For the tested catalyst layer, L1_2_‐Pt_3_Mn@Mn–N–C possesses a more excellent superhydrophobic surface than Pt/C (Figure S35), suggesting a more stable cathode triple phase interface [44]. After AST, the contact angles of L1_2_‐Pt_3_Mn@Mn–N–C and Pt@Mn–N–C show smaller alterations than commercial Pt/C, implying superior catalyst layer stability. This further confirms that the Mn–N–C support effectively inhibits catalyst layer structural degradation, ensuring the formation of a reliable triple phase boundary and rapid mass transportation. Figure 5g also presents the long‐term 0.67 V constant voltage stability evaluation under H_2_‐Air conditions, a typical operating voltage for fuel cells in real‐world applications [45]. After 100 h of continuous operation, L1_2_‐Pt_3_Mn@Mn–N–C demonstrates a 12% decay, while commercial Pt/C shows a 22% decrease, highlighting the comprehensive durability of the proposed catalyst. Notably, a direct comparison with literature and challenging DOE requirements (Figure S36 and Table S10) confirms that L1_2_‐Pt_3_Mn@Mn–N–C in MEA meets the DOE 2025 targets for both catalytic activity and stability, ranking among the top‐performing Pt‐based catalysts to date for fuel cells. This outstanding performance stems from the complementary advantages and synergistic effects between the cubical ordered L1_2_‐Pt_3_Mn intermetallic alloy and the porous Mn–N–C support, which synergistically stabilize the triple phase boundary and enhance mass/charge transfer.

## 3 Conclusions

In summary, through the synergistic design of ordered intermetallic alloys and single‐atom carbon supports, this work has successfully synthesized a high‐performance and durable L1_2_–Pt_3_Mn@Mn–N–C catalyst for oxygen reduction. This approach effectively enhances the atomic ordering degree of the intermetallic alloy and enables uniform deposition near the Mn‐N_4_ centers. The support with abundant Mn single‐atom sites exhibits a well‐developed pore structure and large SSA, which effectively restricts the growth of Pt particles and maintains a small particle size even during high‐temperature alloying. The strong anchoring effect between Pt_3_Mn and the Mn–N–C support suppresses metal leaching, which endows the catalyst with excellent activity and stability in acidic RDE measurements. The fuel cell performance is consistent with electrochemical results. A peak power density of 1.15 W cm^−2^ is achieved in H_2_/Air PEMFCs at a total Pt loading of 0.2 mg_Pt_ cm^−2^. Remarkable PEMFC durability results reveal that after 30 000 potential cycles, the ECSA loss is only 19.4%, the voltage decay at 0.8 A cm^−2^ is 19 mV, and the MA retention reaches 64%, all of which surpass the DOE 2025 targets. After 100 h operation at 0.67 V, the activity degradation is merely 12%, positioning this catalyst among the best‐performing fuel cell cathode catalysts reported to date. The improved activity originates from the high L1_2_ atomic ordering of Pt clusters and the presence of Mn sites in the support, which jointly induce directional interfacial electron transfer and facilitate the rate‐limiting *OH desorption step. In situ ICP‐MS and DFT calculations further confirm that this synergistic interaction gives rise to a strong metal‐support interaction and high dissolution resistance, significantly boosting the long‐term stability of the catalyst. The synergistic design of active species and functional support demonstrated in this work offers a promising route for developing highly robust catalysts for harsh scenarios and can be extended to a wide spectrum of electrochemical energy conversion technologies.

## Author Contributions

**Gongjin Chen**: conceptualization, methodology, validation, investigation, visualization, writing – review and editing, writing – original draft, data curation. **Tianshuai Wang**: methodology, software, writing – original draft, writing – review and editing. **Xiaoyi Qiu**: conceptualization, methodology, writing – original draft, writing – review and editing, data curation. **Shiyuan Liu**: conceptualization, writing – original draft, writing – review and editing. **Cunpu Li**: validation. **Zidong Wei**: validation. **Wei Xing**: validation. **Haijiang Wang**: validation, methodology, supervision. **Minhua Shao**: writing – review and editing, writing – original draft, conceptualization, methodology, funding acquisition, supervision.

## Conflicts of Interest

The authors declare that none of the work reported in this study could have been influenced by any known competing financial interests or personal relationships.

## Supporting information




**Supporting File 1**: anie73097‐sup‐0001‐SuppMat.docx.

## Data Availability

The data that support the findings of this study are available from the corresponding author upon reasonable request.
