# Supplementary material for: Ordered Pt3Mn Intermetallic Nanoparticles Supported on Atomically Dispersed Mn–N–C as Electrocatalysts for Fuel Cells
Source: Angew Chem Int Ed Engl. 2026 Jun 9;65(33):e7818348. doi: 10.1002/anie.7818348 (PMC13452516; doi:10.1002/anie.7818348)
Supplement: Supplementary file 1 [file ANIE-65-e7818348-s001.docx]

Supporting Information

Ordered Pt_3_Mn Intermetallic Nanoparticles Supported on Atomically Dispersed Mn-N-C As Electrocatalysts for Fuel Cells

Gongjin Chen, ^1,2,3,4,‡^ Tianshuai Wang,^1,5,‡^ Xiaoyi Qiu,^1,3,4,‡^ Shiyuan Liu,^1,3,4^ Cunpu Li,^6^ Zidong Wei,^6^ Wei Xing,^7^ Haijiang Wang,^* 2^ Minhua Shao^* 1,3,4,8^

1. Department of Chemical and Biological Engineering, The Hong Kong University of Science and Technology, Kowloon, Hong Kong, 999077 China

2. Department of Mechanical and Energy Engineering, Key Laboratory of Energy Conversion and Storage Technologies, Southern University of Science and Technology, Shenzhen, Guangdong, 518055 China

3. CIAC-HKUST Joint Laboratory for Hydrogen Energy, The Hong Kong University of Science and Technology, Clear Water Bay, Kowloon, Hong Kong, 999077 China

4. Energy Institute, The Hong Kong University of Science and Technology, Clear Water Bay, Kowloon, Hong Kong, 999077 China

5. Xi'an Key Laboratory of Functional Organic Porous Materials, School of Chemistry and Chemical Engineering, Northwestern Polytechnical University, Xi'an, 710072 China

6. State Key Laboratory of Advanced Chemical Power Sources, School of Chemistry and Chemical Engineering, Chongqing University, Chongqing, 400044 China

7. State Key Laboratory of Electroanalytical Chemistry, Jilin Province Key Laboratory of Low Carbon Chemical Power, Changchun Institute of Applied Chemistry, Chinese Academy of Sciences, Changchun, 130022 China

8. Guangzhou Key Laboratory of Electrochemical Energy Storage Technologies, Fok Ying Tung Research Institute, The Hong Kong University of Science and Technology, Guangzhou, 511458 China

^‡^ These authors contributed equally to this work.

# 1. Experimental Information

## Materials

The platinum source was using Hydrogen Hexachloroplatinate (H_2_PtCl_6_·(H_2_O)_6_, ≥99 % ,Sigma-Aldrich). The manganese-based precursors were employing Manganese (II) chloride, tetrahydrate (MnCl_2_**·**4H_2_O ≥98 %, Sinopharm), 2-methylimidazole (≥99 %, Sigma-Aldrich), Zinc nitrate hexahydrate (Zn(NO_3_)_2_**·**6H_2_O, 99.998 %, Aladdin), Ammonium chloride (NH_4_Cl, 99.998 %, Sigma-Aldrich). Proton exchange membrane (Nafion HP, Dupont), ionomer (Nafion D2021,Dupon), commercial Pt/C (Pt loading: 28 %), commercial PtCo/C, and gas diffusion layer (Carbon Paper 22BB, Sigracet) were employed to prepare membrane electrode assemblies (MEA). Methanol (CH_3_OH, 99 %, Amichem), Perchloric acid (HClO_4_, 70 %, Sigma-Aldrich), Isopropanol (IPA, 99.9%, Hexeal) and deionized water (Millipore Milli-Q, ＞18.2 MΩ cm) was adopted to prepare the multiple aqueous solutions in this study.

## Preparation of Mn-N-C

The synthesis of Mn-N-C referenced our previous work.^[1]^ As the example, 2.15 g Zn(NO_3_)_2_·6H_2_O, 50 mg MnCl_2_·4H_2_O and an appropriate amount of 0.1M HCl are evenly dispersed in 22 mL of methanol. After ultrasonic for 60 minutes, solution A is obtained. 40 mL of methanol was slowly added to the three-necked flask containing 12.9 g of 2-methylimidazole, and after ultrasonic dispersion for 60 min, solution B was obtained. Solution A was slowly added dropwise into the three-necked flask containing solution B, and then stirred vigorously for 6 hours with argon gas. The milky white Mn doped ZIF-8 was obtained after washed with methanol three times under centrifugation at 15,000 rpm, and overnight in a vacuum chamber at 60 °C. The precursor was ball milled with a ZrO_2_ ball at 600 rpm with an appropriate amount of MnCl_2_·4H_2_O for 12 h. Ball milling serves as a mild yet efficient mechanical doping strategy, boosting the specific surface area and mesopore volume of the resultant Mn-N-C while retaining the dodecahedral morphology.^[1]^ The mixed precursor was then transferred to a tube furnace and sintered at 1100 °C for one hour filled with nitrogen to remove Zn in the precursor and stabilize the whole carbon framework to obtain Mn-N-C.

## Preparation of Pt@Mn-N-C

Pt nanoparticles were deposited on the Mn-N-C support using ethylene glycol reduction. For example, 200 mg Mn-N-C and 400 mL of 50% concentration ethylene glycol are added to a 500 ml flask, and ultrasonicated under nitrogen protection for 2 h. Then, a certain amount of chloroplatinic acid hexahydrate was slowly added into the flask, keeping the mass ratio of Pt to carrier at 30%, and continued ultrasonic for 1.0 h to obtain a uniformly dispersed suspension. Transfer to an oil bath, stir and heat at 140 °C for four hours, always protected with nitrogen during this period. After cooling to room temperature, the samples were collected by suction filtration and vacuumed at 60 °C overnight to obtain the final Pt@Mn-N-C catalyst.

## Preparation of L1_2_-Pt_3_Mn@Mn-N-C

The L1_2_-Pt_3_Mn@Mn-N-C catalyst was synthesized using a freeze-drying-based method. Typically, 10 mL of deionized water was dropped into a centrifugal tube containing 300 mg of Pt@Mn-N-C and a specific quantity of MnCl_2_‧4H_2_O. The mixture underwent sonication in an ice bath for 2 h and was then completely frozen using liquid nitrogen. The mixture was pre-frozen with liquid nitrogen, then transferred to a vacuum freeze-dryer and dried at −55 °C under vacuum for 24 h. Subsequently, the resulting fine powder was transferred into a quartz boat and heated to 750 °C under 7% H_2_/Ar gas, maintaining this temperature for 2 hours in a tube furnace (with 45 min to reach 750 °C). The catalyst obtained was leached at 0.1M HClO_4_ overnight and then vacuum dried at 60 °C for 12 h to yield the final catalyst. The prepared catalyst is labeled as L1_2_-Pt_3_Mn@Mn-N-C. To comprehensively evaluate the effects of synthesis conditions, researchers prepared a range of L1_2_-Pt_3_Mn@Mn-N-C catalysts by adjusting the quantities of manganese contents, pyrolysis times and temperature, while maintaining consistent procedures.

## Physicochemical characterizations

The Scanning electron microscopy (SEM) images were employed using the JSM-7100 instrument (JEOL). Transmission electron microscopy (TEM) images were applied to the JEM-ARM200F (JEOL), high-angle annular dark field STEM (HAADF-STEM) images were obtained on JEM-ARM200F (JEOL). X-ray photoelectron spectra (XPS) and ultraviolet photoemission spectroscopy (UPS) tests were performed on the AXIS Ultra DLD (Kratos Analytical Ltd) equipped with a hemispherical energy analyzer. The X-ray diffraction (XRD) patterns were carried out on a PW1830 powder X-ray diffractometer (Philips) equipped with a Cu Kα radiation source (λ = 1.54056 Å) and a graphite monochromator. Raman spectroscopy was operated using the InVia system (Renishaw) with a 514 nm light source. The Brunauer–Emmett–Teller (BET) test was analyzed using the BELSOPR MINI X (Microtrac MRB). Inductively coupled plasma mass spectrometry (ICP-MS) was analyzed by 7900 ICP-MS (Agilent) for elemental and quantitative analysis. Contact angle was measured using the contact angle meter (Biolin Theta) for the determination of hydrophilicity.

## Electrochemical measurements

The rotating disk electrode (RDE) test was conducted with CHI 760e electrochemical workstation and Pine Research Instruments RDE system. For the electrocatalyst ink, specifically, 5 mg of the sample is taken, and 4 mL of ultrapure water, 1 mL IPA, and 10 μL of 5 wt% Nafion D2021 are added under ice bath ultrasound. 10 μL of the fully dispersed catalyst ink is taken and dropped onto a glassy carbon electrode with a diameter of 0.5 cm, and it is allowed to dry naturally to remove the solvent and fix the catalyst particles. The final RDE loadings obtained were 0.05 mg cm^-2^ for precious metal samples. A glass fiber carbon electrode dripping with catalyst ink was used as the working electrode, the counter electrode was a carbon rod, and the reference electrode was Ag/AgCl for 0.1 M HClO_4_ solution. All reference electrodes were calibrated against an in-house reversible hydrogen electrode (RHE) prior to testing, with a Pt wire in H₂-saturated electrolyte. The measured potential differences were used to convert raw data to the RHE scale. For 0.1 M HClO₄, E_RHE_=E_Ag/AgCl_+0.273 V.

Cyclic Voltammetry (CV) test was performed in two three-electrode cells with a scan range from 0.05 to 1.2 V vs. RHE, scan rate 50 mV s^-1^. The ORR performance test, linear sweep voltammogram (LSV), is conducted in a three-electrode system of saturated oxygen, with a rotation speed of 1600 rpm, a scan rate of 10 mV s^-1^, and a scanning range of 0 to 1.0 V vs. RHE. The square wave potential accelerated aging test (AST) of the sample is conducted under saturated oxygen, the potential changes are 0.6 and 1.0 V, the duration of each voltage point is 3s, and the scan rate is 5 mV s^-1^. The electrochemically active area (ECSA) of the catalyst is calculated by analyzing the CV curve get.

$$ECSA=\frac{Q_{r}}{C*m_{Pt}}$$

Where Q_r_ is the hydrogen adsorption charge during CV test, C is the charge of full monolayer coverage of H atoms onto clean polycrystalline Pt (210 μC cm^-2^), and m_Pt_ is the catalyst loading on the surface of the glassy carbon electrode. The rotating ring disk electrode (RRDE) technique was applied to obtain the peroxide (HO_2_^-^) yield and the electron transfer number (n) during ORR process. The RRDE is composed of a glassy carbon disk electrode (diameter = 0.5 cm) and a concentric Pt ring with an inner diameter of 0.5 cm and an outer diameter of 0.7 cm. The catalyst ink formulation and drop method are consistent with the above RDE. The ring current measured in N_2_-saturated 0.1 M HClO_4_ is taken as the background current, which is subtracted from the ring current obtained in O_2_-saturated electrolyte. This correction ensures the accurate calculation of H_2_O_2_ yield and electron transfer number. Polarization curves were recorded at the electrode rotation speed of 1600 rpm with a potential scan rate of 10 mV s^-1^, and the ring electrode potential was kept constant at 1.1 V. Prior to testing, the Pt ring was electrochemically cleaned by cycling in 0.1 M HClO₄ between 0.05–1.5 V vs. RHE for 50 cycles at 100 mV s⁻¹ until a stable curve was obtained. The H_2_O_2_ yield and the transferred electron number (n) of the ORR were calculated via using the following:

$$H_{2}O_{2}\left( \% \right)=\frac{200{Ir}/N}{{I_{r}}/{N+I_{d}}}$$

$$n=\frac{{4I}_{d}}{{I_{r}}/N+I_{d}}$$

In which I_r_ and I_d_ are ring and disk current; the current collection efficiency of the Pt ring (N) is 0.38. Tafel slopes were calculated as follows:

$$\eta=a+blog(\frac{j}{j_{0}})$$

in which η, b, j, and j_0_ are the overpotential, the Tafel slope, the current density and the exchange current density, respectively. Besides, the numerical values are rounded to match the inherent precision of the measurement technique. For example, Tafel slopes are reported to the nearest integer (mV dec^-1^), and BET surface areas are given as ±1 m^2^ g^-1^.

## In-situ ICP-MS measurements

A homemade stopper equipped with a probe tip was used during the in situ ICP-MS testing. A PFA capillary tube as a stationary probe was positioned close to the disk electrode, ensuring that the flow direction of the electrolyte was vertical to the normal direction of the electrode surface. The probe position was kept the same during every test to avoid the distance discrepancy. The electrolyte was driven out with a pump, where the probe capillary was joined to a tube and delivered to the ICP-MS inlet. During the testing, the plasma power was set at 1600 W. During the dissolution monitoring tests, the flow speed was adjusted to decrease the time from the dissolve position to the detection system. Five Pt and Mn standard solutions were used to calibrate the relation between the signal intensities from the ICP-MS response and the corresponding concentration. A CV protocol triggered the Pt and Mn metal dissolution at a sweep speed of 5 mV s^−1^ in the 0.05 to 1.2 V potential range. Furthermore, the Mn and Pt standard curve was calibrated before each experiment to quantify the Mn and Pt dissolution amount. Other test conditions are consistent with RDE measurements.

## Computational Details

Spin-polarized density functional theory calculations were conducted using the Vienna Ab-initio Simulation Package with the projector augmented wave (PAW) formalism.^[2]^ The Gaussian smearing method was used and the width of smearing was chosen as 0.05 eV. The energy cutoff for plane-wave expansion of the PAWs was set to 400 eV. To decouple the periodic replicas, a vacuum layer of approximately 15 Å in thickness was inserted in the vertical direction. The DFT-D3 functional was applied to consider the effect of the physical van der Waals interaction in all calculated simulations.^[3]^ The structure relaxation was continued until the forces and energies on all the atoms were converged to less than 0.01 eV/Å and 1×10^-5^ eV. The computational hydrogen electrode (CHE) model introduced by Nørskov and co-workers was used to calculate the Gibbs free energy of reactions in involving electrons/proton transfer. The oxygen reduction reaction (ORR) in an acidic electrolyte proceeds through the following elementary steps ^[4]^

*O_2_* (g) + H^+^ + e^−^ → OOH**

*OOH* + H^+^ + e^−^ → O* +H_2_O (I)*

*O* + H^+^ + e^−^ → OH**

*OH* + H^+^ + e^−^ → H_2_O (I) + **

Where * denotes the active site on the catalyst, OOH, O, and OH* refer to the adsorbed intermediates involved in the reaction. The (l) and (g) notations represent the liquid and gas phases, respectively.

## MEA preparation and tests

The MEA samples in this article are catalyst coated membrane (CCM) types in which catalyst ink is sprayed on the membrane by a vacuum spraying machine (SONOTEK). For the catalyst ink, take a certain amount of catalyst and add deionized water, IPA and ionomer in sequence, and continuously sonicate in an ice bath for 6 hours to obtain the catalyst ink. The base plate temperature during spraying is 80 °C. The HOR catalyst of the anode is Pt/C TKK, and the cathode ORR catalyst is the sample synthesized in this study. The catalyst loading for the prepared CCM is Anode: 0.05 mg_pt_ cm^−2^, Cathode: 0.15 mg_pt_ cm^−2^. The sprayed CCM needs to be sandwiched between two pieces of 22BB carbon paper and 400 lbf hydraulic pressure for three minutes under preheating at 140 °C. The final MEA includes PEM, CL and GDL. Before formal testing, the MEA and two glass fiber gaskets were sandwiched between two graphite bipolar plates with a dense serpentine flow field and clamped with 8 screws at a torque of 12 Nm to obtain an assembled single cell.

Polarization curve is a key evaluation index of MEA performance. The cell temperature is stable at 80 °C, the stoichiometric ratio of anode (hydrogen) and cathode (pure oxygen or air) is 2.0/3.5, the relative humidity is 100 %, and the back pressure is 150 kPa. Use constant voltage mode with a voltage sweep range of 1.2 to 0.2 V and collect single cell currents every 30 s. The principle of ECSA measurement in MEA is the same as that of RDE. The anode gas was first switched to argon with 5% H2 to mitigate hydrogen cross-over effects during CV testing. In a single cell, the cathode side is the working electrode, the anode is the counter electrode and the reference electrode, the scanning voltage range of CV is 0-1.2 V, and the scanning rate is 50 mV s^-1^, and the relative humidity is 150 %. EIS data was collected under the same conditions as the polarization curves to evaluate the relative contributions of charge transfer resistance and mass transfer resistance to the net cell voltage loss. The frequency range was 10000 Hz to 0.1 Hz, the amplitude was 20 mV, and 10 data points were recorded per decade of frequency change. The AST durability test of PEMFC follows DOE standards: For the electrocatalyst itself, using the square wave potentials of 0.6 V and 0.95V, and each voltage point is stable for 3 seconds. During the voltage cycle, the anode and cathode gas supply conditions are H_2_/N_2_=100/80 sccm, the relative humidity is 100 %, the back pressure is 150 kPa, and the cell temperature is stable at 80 °C.

# 2. Supplementary Figures

Figure S1. SEM image of Mn-doped ZIF-8.

Figure S2. Detailed schematic illustration of the synthetic process for the L1_2_-Pt_3_Mn intermetallic nanoparticles supported by Mn-N-C.

Figure S3. SEM image of the L1_2_-Pt_3_Mn@Mn-N-C.

Figure S4. Typical TEM images of (a) commercial Pt/C, prepared (b) Pt@Mn-N-C and (c) L1_2_-Pt_3_Mn@Mn-N-C.

Figure S5. (a) TEM image and (b) corresponding particle size distribution of Pt/C.

Figure S6. (a) TEM image and (b) corresponding particle size distribution of Pt@Mn-N-C.

Figure S7. (a) Selected area electron diffraction (SAED) pattern, and (b) FFT pattern ( of L1_2_-Pt_3_Mn@Mn-N-C.

Figure S8. XRD patterns of the L1_2_-Pt_3_Mn@Mn-N-C catalyst with different pyrolysis temperatures.

Figure S9. The ordering degree by XRD patterns of the L1_2_-Pt_3_Mn@Mn-N-C catalyst with different pyrolysis temperatures, according to the (110)/(111) intensity ratio.^[5]^

Figure S10. XRD patterns of the L1_2_-Pt_3_Mn@Mn-N-C catalyst with different pyrolysis time, while temperature maintain 750 ℃.

Figure S11. The pore size distribution based on the BJH method of L1_2_-Pt_3_Mn@Mn-N-C, Pt@Mn-N-C, commercial Pt/C and Mn-N-C catalysts.

Figure S12. XPS spectrum of wide survey for L1_2_-Pt_3_Mn@Mn-N-C, Pt@Mn-N-C, and Mn-N-C catalysts.

Figure S13. XPS spectrum of C 1s for L1_2_-Pt_3_Mn@Mn-N-C, Pt@Mn-N-C, and Mn-N-C catalysts.

Figure S14. XPS spectrum of (a) Pt 4f and (b) Mn 2p for L1_2_-Pt_3_Mn@Mn-N-C catalysts with various pyrolysis temperature.

Figure S15. Secondary electronic cutoff energy (E_cutoff_) and work function for L1_2_-Pt_3_Mn@Mn-N-C and Pt@Mn-N-C. Work function (Φ) = 21.22 eV-E_cutoff_, 21.22 eV is the photon energy of the He (I) I excitation source.

Figure S16. Tafel slope of L1_2_-Pt_3_Mn@Mn-N-C, Pt@Mn-N-C, and commercial Pt/C.

Figure S17. Ring current of L1_2_-Pt_3_Mn@Mn-N-C, Pt@Mn-N-C, and commercial Pt/C.

Figure S18. The steady state (a) LSV and (b) CV curves of L1_2_-Pt_3_Mn@Mn-N-C with various pyrolysis temperature. At a rotating speed of 1600 rpm in 0.1 M HClO_4_ solution.

Figure S19. CV curves before and after 30k or 60k AST tests via square-wave potential cycling from 0.6 to 0.95 V in N_2_-saturated 0.1 M HClO_4_ solution: (a) L1_2_-Pt_3_Mn@Mn-N-C, (b) Pt@Mn-N-C, and (c) commercial Pt/C. The ECSA is calculated from the hydrogen adsorption charge.

Figure S20. XPS spectrum of (a) Pt 4f and (b) Mn 2p, and (c) N 1s for L1_2_-Pt_3_Mn@Mn-N-C catalysts before and after AST tests via square-wave potential 30k cycling from 0.6 to 0.95 V.

Figure S21. Experiential determination of the relationship between signal intensities from ICP-MS and corresponding concentration. (a) The equation for Pt concentration in the range of 0-100 ppb, and (b) the equation for Mn concentration in the range of 0-100 ppb.

Figure S22. Dissolution rates of Pt for (a) L1_2_-Pt_3_Mn@Mn-N-C, (b) Pt@Mn-N-C, (c) commercial PtCo/C, and (d) Pt/C recorded during the 0.05-1.2 V potential cycle in 0.1 M HClO_4_ solution at 5 mV s^-1^.

Figure S23. Dissolution rates of Mn for (a) L1_2_-Pt_3_Mn@Mn-N-C, and (b) Pt@Mn-N-C recorded during the 0.05-1.2 V potential cycle in 0.1 M HClO_4_ solution at 5 mV s^-1^.

Figure S24. Atomic configurations of (a) (b) Pt_4_/Graphene, and distal atomic models of (c) (d) Pt_4_/SAMn, (e) (f) Pt_3_Mn/SAMn.The gray, gold, blue, and green represent the C, Mn, Pt, and N atoms, respectively.

Figure S25. DFT calculated binding energy of Pt_3_Mn/SAMn, Pt_4_/SAMn, and Pt_4_/Graphene.

Figure S26. Proposed adsorption configurations of the intermediates with relaxed adsorption structures on (a) Pt_4_/SAMn, and (b) Pt_4_/Graphene during the ORR processes.

Figure S27. Crystal orbital Hamilton population (COHP) analysis of Pt-O(OH) bonds for Pt_4_/Graphene.

Figure S28. Fuel cell performance of L1_2_-Pt_3_Mn@Mn-N-C catalyst with different I/C ratio. (a) Polarization curves, power densities, and (b) corresponding Nyquist plot at 0.1 A cm^-2^.

Figure S29. Polarization curves and corresponding power densities of L1_2_-Pt_3_Mn@Mn-N-C catalyst under different operating relative humidity.

Figure S30. Nyquist plots at 0.1 A cm^-2^ of (a) L1_2_-Pt_3_Mn@Mn-N-C, (b) Pt@Mn-N-C, and (c) commercial Pt/C before and after 30k square-wave potential cycling from 0.6 to 0.95 V in MEA. Equivalent circuit in EIS fitting, R_ohm_ is the resistor for the ohmic resistance of PEMFC, R_ct_ is the resistor for the charge transfer resistance, and CPE1 is the constant phase element for the double-layer capacitance.

Figure S31. Nyquist plots at 1.5 A cm^-2^ of (a) L1_2_-Pt_3_Mn@Mn-N-C, (b) Pt@Mn-N-C, and (c) commercial Pt/C before and after 30k square-wave potential cycling from 0.6 to 0.95 V in MEA. Equivalent circuit in EIS fitting, R_ohm_ is the resistor for the ohmic resistance of PEMFC, R_ct_ is the resistor for the charge transfer resistance, and CPE1 is the constant phase element for the double-layer capacitance.

Figure S32. AC-STEM image of L1_2_-Pt_3_Mn@Mn-N-C catalyst after 30,000 cycles in MEA.

Figure S33. Morphological and structural characterization of L1_2_-Pt_3_Mn@Mn-N-C catalyst under after 30,000 cycles in MEA. (a) TEM image with corresponding particle size distribution, (b) XRD patterns, (c) high-resolution TEM image and (d) SAED pattern.

Figure S34. Morphological characterization of L1_2_-Pt_3_Mn@Mn-N-C catalyst under after 30,000 cycles in MEA. (a) HAADF-STEM image, and corresponding element mapping (b) mix, (c) Pt, and (d) Mn.

Figure S35. Contact angle of (a-b) commercial Pt/C, (c-d) Pt@Mn-N-C, and (e-f) L1_2_-Pt_3_Mn@Mn-N-C before and after 30k square-wave potential cycling from 0.6 to 0.95 V in MEA

Figure S36. Graphical achievement of ORR performance at MEA level for L1_2_-Pt_3_Mn@Mn-N-C electrocatalysts compared to the activity and durability of the 2025 DOE targets.

# 3. Supplementary Tables

**Table S1.** Pt and Mn content in prepared L1_2_-Pt_3_Mn@Mn-N-C, Pt@Mn-N-C, and Mn-N-C determined by ICP-MS. The Mn content in Pt_3_Mn nanoparticles = total Mn content - monatomic Mn content.

| Samples | Pt (wt%) | Mn (wt%) |
| --- | --- | --- |
| Pt_3_Mn@Mn-N-C | 29.72 | 4.41 |
| Pt@Mn-N-C | 29.91 | 1.61 |
| Mn-N-C | / | 1.62 |
| Pt_3_Mn nanoparticles | 29.72 | 2.79 |

**Table S2.** BET surface areas and pore distribution of prepared L1_2_-Pt_3_Mn@Mn-N-C, Pt@Mn-N-C, Mn-N-C, and commercial Pt/C.

| Samples | V_micro_ | | V_meso_ | | V_macro_ | | V_total_ | S_BET_ |
| --- | --- | --- | --- | --- | --- | --- | --- | --- |
|  | cm^3^ g^-1^ | % | cm^3^ g^-1^ | % | cm^3^ g^-1^ | % | cm^3^ g^-1^ | m^2^ g^-1^ |
| Pt_3_Mn@Mn-N-C | 0.3237 | 34.14% | 0.4832 | 50.96% | 0.1413 | 14.90% | 0.9482 | 648.55 |
| Pt@Mn-N-C | 0.4089 | 43.57% | 0.4476 | 47.70% | 0.0819 | 8.73% | 0.9384 | 624.9 |
| Mn-N-C | 0.2156 | 23.11% | 0.4223 | 45.27% | 0.2849 | 30.54% | 0.9328 | 1256.4 |
| Pt/C | 0.2913 | 31.65% | 0.3654 | 39.70% | 0.2637 | 28.65% | 0.9204 | 460.4 |

**Table S3.** Elemental quantification (at.%) determined by XPS for prepared L1_2_-Pt_3_Mn@Mn-N-C, Pt@Mn-N-C, Mn-N-C.

| Samples | C | N | Pt | Mn | Zn |
| --- | --- | --- | --- | --- | --- |
| Pt_3_Mn@Mn-N-C | 70.32 | 2.27 | 4.41 | 1.76 | 0.01 |
| Pt@Mn-N-C | 88.33 | 3.30 | 2.41 | 0.31 | 0.01 |
| Mn-N-C | 95.18 | 2.69 | / | 0.27 | 0.01 |

**Table S4.** Elemental quantification (at.%) determined by XPS for prepared L1_2_-Pt_3_Mn@Mn-N-C before and after 30,000 cycles square wave test.

| Samples | C | N | Pt | Mn | Zn |
| --- | --- | --- | --- | --- | --- |
| BOL | 70.32 | 2.27 | 4.41 | 1.76 | 0.01 |
| EOL | 70.94 | 2.11 | 4.33 | 1.69 | 0.01 |

**Table S5.** Summary of previously reported Pt-based catalysts in terms of their RDE activity and stability in 0.1 M HClO_4_ solution

| Catalyst | RDE activity | | | RDE Stability | | References |
| --- | --- | --- | --- | --- | --- | --- |
|  | E_1/2_  (V *vs.* RHE) | MA@0.9V  (A mg_Pt_^−1^) | ESCA  (m^2^ g_Pt_^−1^) | E_1/2_  (V *vs.* RHE) | MA retention  (%) |  |
| L1_2_-Pt_3_Mn@Mn-N-C | 0.905 | 0.45 | 46.1 | 0.892 (30k)  0.866 (60k) | 83.9 (30k) | This work |
| i-CoPt@Pt/KB | 0.9 | 2.1 | 58 | 0.89 | 85.7 | ^[6]^ |
| L1_0_-N-PtCo-H@Pt/C | 0.895 | 1.22 | 55 | - | - | ^[7]^ |
| Pt_56_Mn_44_/C | 0.91 | 0.53 | 53 | 0.896 (10k) | 96 (10k) | ^[8]^ |
| P-L1_0_-PtFeGa_0.1_/C | 0.926 | 1.5 | 76.7 | 0.902 | 88.9 | ^[9]^ |
| L1_2_-Pt_3_Co@Mn_SA_-NC | 0.955 | 0.19 | 77.5 | 0.945 | 84 | ^[10]^ |
| Surf-IM-PtFe NWs/C | 0.92 | 0.49 | 29 | 0.917 | 87.8 | ^[11]^ |
| PtCo/P_2.73_O_x_–KB | 0.928 | 1.1 | 68 | 0.915 | 70.9 | ^[12]^ |
| PtFe/NcPS | 0.91 | 1.3 | 116 | 0.89 (10K) | 85.7 (10K) | ^[13]^ |
| O-PtCo@GCoNC | 0.90 | 0.92 | - | 0.88 | 86.8 | ^[14]^ |
| Pt_1_Fe_1_/Fe_1_-N-C | 0.93 | 0.92 | - | 0.9 (10K) | - | ^[15]^ |
| PtCo@NGCS/KB-800 | 0.90 | 0.81 | 89 | 0.83 | 71.6 | ^[16]^ |
| L1_0_-PtCoIn@Pt | 0.92 | 1.22 | 82 | 0.89 | 95 | ^[17]^ |
| DC-Pt_1_Co_1_/CNT | 0.94 | 2.2 | 88.1 | 0.92 | 90 | ^[18]^ |
| O-PtCo-FeNC | 0.89 | 1.32 | - | 0.85 | 68.1 | ^[19]^ |
| Co/N/C@PtCo-NPs | 0.878 | 0.26 | 91 | 0.835 | 88.4 | ^[20]^ |

**Table S6.** DFT Calculated total energy at the proximal end and distal end of the Pt_3_Mn/SAMn and Pt_4_/SAMn models

| Model | Pt_3_Mn/SAMn | Pt_4_/SAMn |
| --- | --- | --- |
| Proximal end | -472.88 eV | -468.40 eV |
| Distal end | -471.89 eV | -467.43 eV |

**Table S7.** DFT Calculated free energy of various ORR species: Pt_3_Mn/SAMn, Pt_4_/SAMn and Pt_4_/Graphene at U = 1.23 V (vs. RHE)

| Reaction Pathway |  | Free Energy (eV) |  |
| --- | --- | --- | --- |
|  | Pt_3_Mn/SAMn | Pt_4_/SAMn | Pt_4_/Graphene |
| OOH* | -0.635 | -0.815 | -0.855 |
| O* | -1.22 | -1.42 | -1.68 |
| OH* | -1.135 | -1.215 | -1.395 |

**Table S8.** Fitted EIS results of (a) L1_2_-Pt_3_Mn@Mn-N-C, (b) Pt@Mn-N-C, and (c) commercial Pt/C before and after 30k square-wave potential cycling from 0.6 to 0.95 V in MEA at 0.1 A cm^-2^.

| Samples | | R_ohm_ | R_ct_ |
| --- | --- | --- | --- |
| Pt_3_Mn@Mn-N-C | BOL | 0.233 | 0.788 |
|  | EOL | 0.237 | 0.871 |
| Pt@Mn-N-C | BOL | 0.231 | 0.802 |
|  | EOL | 0.236 | 0.983 |
| Pt/C | BOL | 0.232 | 0.891 |
|  | EOL | 0.238 | 1.177 |

**Table S9.** Fitted EIS results of (a) L1_2_-Pt_3_Mn@Mn-N-C, (b) Pt@Mn-N-C, and (c) commercial Pt/C before and after 30k square-wave potential cycling from 0.6 to 0.95 V in MEA at 1.5 A cm^-2^.

| Samples | | R_ohm_ | R_ct_ |
| --- | --- | --- | --- |
| Pt_3_Mn@Mn-N-C | BOL | 0.227 | 0.573 |
|  | EOL | 0.231 | 0.642 |
| Pt@Mn-N-C | BOL | 0.226 | 0.572 |
|  | EOL | 0.232 | 0.644 |
| Pt/C | BOL | 0.224 | 0.575 |
|  | EOL | 0.235 | 0.848 |

**Table S10.** Summary of previously reported Pt-based catalysts in terms of their fuel cell performance and durability.

| Catalyst | Pt loading in MEA  (mg_Pt_ cm^−2^) | P_Max_ @ H_2_/Air  (W cm^−2^) | | MA@0.9V  (A mg_Pt_^−1^) | | ECSA loss  (%) | Voltage loss at 0.8 A cm^-2^ (mV) | References |
| --- | --- | --- | --- | --- | --- | --- | --- | --- |
|  |  | BOL | EOL | BOL | EOL |  |  |  |
| L1_2_-Pt_3_Mn@Mn-N-C | A: 0.05  C: 0.15 | 1.15 | 1.06 (30k)  0.97 (60k) | 0.69 | 0.44 | 19.4 | 19 | This work |
| i-CoPt@Pt/KB | A: 0.10  C: 0.10 | 1.28* | 1.14 | 0.53 | 0.34 | 19 | 29 | ^[6]^ |
| L1_0_-CoPt@Pt-shell | A: 0.10  C: 0.10 | 0.99 | 0.87 | 0.60 | 0.31 | 33 | 26 | ^[21]^ |
| L1_0_-N-PtCo-H@Pt/C | A: 0.05  C: 0.10 | 0.98 | 0.76 | 0.88 | 0.62 | 9 | 22 | ^[7]^ |
| Pt_56_Mn_44_/C | A: 0.10  C: 0.10 | 1.06 | 0.795 | 0.52 | 0.35 | 23 | 25 | ^[8]^ |
| P-L1_0_-PtFeGa_0.1_/C | A: 0.10  C: 0.07 | 0.99 | 0.83 | 0.84 | 0.60 | 16 | 11 | ^[9]^ |
| L1_2_-Pt_3_Co@Mn_SA_-NC | A: 0.10  C: 0.10 | 1.31 | 1.09 | 0.91 | 0.45 | 9 | 40 | ^[10]^ |
| Surf-IM-PtFe NWs/C | A: 0.10  C: 0.10 | 1.00* | 0.76 | 0.47 | 0.369 | 10 | 31 | ^[11]^ |
| PtCo/P_2.73_O_x_–KB | A: 0.10  C: 0.10 | 1.2 | 0.9 | 0.61 | 0.48 | - | 10 | ^[12]^ |
| PtFe/NcPS | A: 0.05  C: 0.10 | 1.11 | 0.88 | 0.77 | 0.51 | 12 | 58 | ^[13]^ |
| O-PtCo@GCoNC | A: 0.10  C: 0.12 | 1.04 | 0.95 | - | - | - | 28 | ^[14]^ |
| Pt_1_Fe_1_/Fe_1_-N-C | A: 0.10  C: 0.10 | 0.88 | 0.76 | 0.51 | 0.39 | - | 45 | ^[15]^ |
| PtCo@NGCS/KB-800 | A: 0.10  C: 0.15 | 0.60 | 0.56 | - | - | 10.1 | 46 | ^[16]^ |
| L1_0_-PtCoIn@Pt | A: 0.10  C: 0.10 | 0.98 | 0.76 | 0.46 | 0.27 | - | 47 | ^[17]^ |
| DC-Pt_1_Co_1_/CNT | A: 0.10  C: 0.10 | 1.18 | 1.04 | 1.40 | 1.19 | - | 11 | ^[18]^ |
| DOE target |  |  |  | ≥0.44 | ≥0.264 | ≤40% | ≤30 |  |

* Back pressure is 250 kPa. Other unlabeled results were obtained at a back pressure of 150 kPa, 100% RH, and 80 ^o^C.

# References

[1]	G. Chen, X. Qiu, S. Liu, Y. Cui, Y. Sun, Y. Zhang, Y. Liu, G. Liu, Y. Kim, W. Xing, H. Wang, M. Shao, *Angew. Chem. Int. Ed.* **2025**, *64*, e202503934.

[2]	aG. Kresse, J. Furthmüller, *Phys. Rev. B* **1996**, *54*, 11169-11186; bP. E. Blöchl, *Phys. Rev. B* **1994**, *50*, 17953-17979.

[3]	J. Moellmann, S. Grimme, *The Journal of Physical Chemistry C* **2014**, *118*, 7615-7621.

[4]	J. K. Nørskov, J. Rossmeisl, A. Logadottir, L. Lindqvist, J. R. Kitchin, T. Bligaard, H. Jónsson, *J. Phys. Chem. B* **2004**, *108*, 17886-17892.

[5]	aY. Xiong, Y. Yang, H. Joress, E. Padgett, U. Gupta, V. Yarlagadda, D. N. Agyeman-Budu, X. Huang, T. E. Moylan, R. Zeng, A. Kongkanand, F. A. Escobedo, J. D. Brock, F. J. DiSalvo, D. A. Muller, H. D. Abruña, *Proceedings of the National Academy of Sciences* **2019**, *116*, 1974-1983; bZ. Cui, H. Chen, M. Zhao, F. J. DiSalvo, *Nano Lett.* **2016**, *16*, 2560-2566.

[6]	T. Y. Yoo, J. Lee, S. Kim, M. Her, S.-Y. Kim, Y.-H. Lee, H. Shin, H. Jeong, A. K. Sinha, S.-P. Cho, Y.-E. Sung, T. Hyeon, *Energy Environ. Sci.* **2023**, *16*, 1146-1154.

[7]	M. I. Maulana, T. H. Jo, H.-Y. Lee, C. Lee, C. Gyan-Barimah, C.-H. Shin, J.-H. Yu, K.-S. Lee, S. Back, J.-S. Yu, *J. Am. Chem. Soc.* **2024**, *146*, 30922-30932.

[8]	Y. Nie, Y. Sun, B. Song, Q. Meyer, S. Liu, H. Guo, L. Tao, F. Lin, M. Luo, Q. Zhang, L. Gu, L. Yang, C. Zhao, S. Guo, *Angew Chem Int Ed Engl* **2024**, *63*, e202317987.

[9]	Y. Li, J. Liang, Z. Lin, X. Liu, S. Li, X. Yan, Z. Cai, J. Han, Y. Zhao, T. Wang, Q. Li, *Renewables* **2024**, *2*, 223-232.

[10]	Y. Zeng, J. Liang, C. Li, Z. Qiao, B. Li, S. Hwang, N. N. Kariuki, C.-W. Chang, M. Wang, M. Lyons, S. Lee, Z. Feng, G. Wang, J. Xie, D. A. Cullen, D. J. Myers, G. Wu, *J. Am. Chem. Soc.* **2023**, *145*, 17643-17655.

[11]	Y. Ma, J. Peng, J. Tian, W. Gao, J. Xu, F. Li, P. Tieu, H. Hu, Y. Wu, W. Chen, L. Pan, W. Shang, P. Tao, C. Song, H. Zhu, X. Pan, T. Deng, J. Wu, *Sci. Adv.* **2024**, *10*, eado4935.

[12]	S.-N. Hu, W.-C. Xu, N. Tian, S.-M. Chen, M.-Y. Li, J.-F. Shen, J.-X. Lin, S.-L. Guo, X.-Y. Huang, Z.-Y. Zhou, S.-G. Sun, *Energy Environ. Sci.* **2024**, *17*, 3099-3111.

[13]	S. Shin, E. Lee, J. Nam, J. Kwon, Y. Choi, B. J. Kim, H. C. Ham, H. Lee, *Adv. Energy Mater.* **2024**, *14*, 2400599.

[14]	B. Wu, H. Yang, L. Li, X. Tang, Y. Wu, B. Huang, D. Lützenkirchen-Hecht, M. Qiu, K. Yuan, Y. Chen, *Adv. Mater.* **2025**, *37*, 2500096.

[15]	F. Zhou, Y. Ruan, M. Zhu, X. Gao, W. Guo, X. Liu, W. Wang, M. Chen, G. Wu, T. Yao, H. Zhou, Y. Wu, *Small* **2023**, *19*, 2302328.

[16]	K. Wan, J. Wang, J. Zhang, B. Li, M. Chai, P. Ming, C. Zhang, *Chem. Eng. J.* **2024**, *482*, 149060.

[17]	L. Zhao, Z. Zhu, J. Wang, J. Zuo, H. Chen, X. Qi, X. Niu, J. S. Chen, R. Wu, Z. Wei, *Angew. Chem. Int. Ed.* **2025**, *64*, e202501805.

[18]	L. Tian, X. Gao, M. Zhu, Z. Huang, B. Wu, C. Chen, X. Ma, Y. Ruan, W. Guo, X. Meng, H. Wang, W. Du, S. He, H. Pan, X. Zheng, Z. Wu, H. Zhou, J. Xia, Y. Wu, *Adv. Mater.* **2025**, *37*, 2417095.

[19]	K. Chen, J. Huang, J. Chen, J. Gao, Z. Lu, X. Liu, S. Lan, G. Jia, S. Ci, Z. Wen, *Energy Environ. Sci.* **2025**, *18*, 6732-6743.

[20]	Y. Zhou, J. Chen, Z. Huang, Y. Peng, L. Xing, C. Tang, N. Wang, L. Meng, M. Wu, L. Du, S. Ye, *Nanoscale* **2024**, *16*, 5215-5221.

[21]	Y.-T. Pan, D. Li, S. Sharma, C. Wang, M. J. Zachman, E. C. Wegener, A. J. Kropf, Y. S. Kim, D. J. Myers, A. A. Peterson, D. A. Cullen, J. S. Spendelow, *Chem Catal.* **2022**, *2*, 3559-3572.
